# Risk Factors Related to Low Ankle-Brachial Index Measured by Traditional and Modified Definition in Hypertensive Elderly Patients

**DOI:** 10.1155/2012/163807

**Published:** 2012-06-07

**Authors:** Raphael Monteiro, Renata Marto, Mario Fritsch Neves

**Affiliations:** Department of Clinical Medicine, State University of Rio de Janeiro, Avenida 28 de Setembro 77, Sala 329, 20551-030 Rio de Janeiro, RJ, Brazil

## Abstract

Peripheral arterial disease (PAD) increases with age and ankle-brachial index (ABI) ≤ 0.9 is a noninvasive marker of PAD. The purpose of this study was to identify risk factors related to a low ABI in the elderly using two different methods of ABI calculation (traditional and modified definition using lower instead of higher ankle pressure). A cross-sectional study was carried out with 65 hypertensive patients aged 65 years or older. PAD was present in 18% of individuals by current ABI definition and in 32% by modified method. Diabetes, cardiovascular diseases, metabolic syndrome, higher levels of systolic blood pressure and pulse pressure, elevated risk by Framingham Risk Score (FRS), and a higher number of total and antihypertensive drugs in use were associated with low ABI by both definitions. Smoking and LDL-cholesterol were associated with low ABI only by the modified definition. Low ABI by the modified definition detected 9 new cases of PAD but cardiovascular risk had not been considered high in 3 patients when calculated by FRS. In conclusion, given that a simple modification of ABI calculation would be able to identify more patients at high risk, it should be considered for cardiovascular risk prediction in all elderly hypertensive outpatients.

## 1. Introduction

 The number of elderly individuals has been progressively increased in the last decades, and it is expected to reach 21% of the world population by 2025 [1]. As elderly people grow, significant changes are observed in the mortality profile for cardiovascular diseases, and it is well known that older subjects have been undertreated [2]. In fact, it is very important to identify a subset of high-risk aged patients that should receive a more aggressive treatment. Many strategies have been developed for this purpose, including the screening for peripheral arterial disease (PAD). PAD increases with age [3, 4] and can be assessed by ankle-brachial index (ABI), a simple and noninvasive test, indicated by the ratio of ankle to brachial systolic blood pressure. Several studies have reported that ABI ≤ 0.9 is associated with an increased risk of death, total cardiovascular disease (CVD), coronary heart disease (CHD), congestive heart failure, stroke, functional decline, and dementia [5–14]. A low ABI is also predictive of target organ damage in hypertension and should be incorporated into routine cardiovascular screening of hypertensive patients [15–17].

There are different formulas to calculate ABI based on the highest or the lowest level of ankle pressure [18, 19]. The current guidelines of the American Heart Association (AHA) and the Inter-Society Consensus for the Management of Peripheral Arterial Disease (TASC II) use the highest ankle pressure as standardization [20, 21]. However, these recommendations are not widely accepted since they could underestimate the true prevalence of PAD [19, 22].

The purpose of the present study was to assess risk factors related to a low ABI in elderly people with hypertension, measured by the current and modified definitions. Recognizing heterogeneity and different profiles among outpatient and institutionalized old patients [10], our study focused on those independent for activities of daily living (ADL).

## 2. Methods

### 2.1. Study Population

This cross-sectional study included patients admitted to the Outpatient Clinic of Hypertension at State University of Rio de Janeiro in a 6-month period. Inclusion criteria were age ≥65 years and previous diagnosis of essential hypertension. Exclusion criteria were significant dependence for ADL, major cognitive deficit, conditions that precluded obtaining ABI (edema, refusing), and ABI higher than 1.4. Eleven patients were excluded at the final evaluation, and a total of 65 elderly hypertensive patients were included. The reasons for exclusion were missing visits (*n* = 3), great cognitive deficit by mini-mental status exam (MMSE) (*n* = 2), leg edema (*n* = 2), ABI > 1.4 (*n* = 1), normal blood pressure (BP) (*n* = 1), significant dependence (*n* = 1), and refusing (*n* = 1). The study protocol was approved by the local ethics committee, and all patients gave informed consent.

### 2.2. Clinical Evaluation

Firstly, participants included in this study had confirmed their diagnosis of hypertension. Patients had at least two measurements of brachial BP and were submitted to a geriatric evaluation that intended to verify cognitive status and independence for ADL. Cognitive deficit was defined as an MMSE score lower than 23 for literate or lower than 18 for illiterate subjects [23, 24]. Significant dependence for ADL was defined by Katz index of independence F and G or as a score lower than 3 [25].

Metabolic syndrome was defined according to the current Brazilian guidelines on metabolic syndrome [26], which includes the presence of three or more of the following: waist circumference > 102 cm (men) or > 88 cm (women), BP ≥ 130/85 mmHg or treatment for hypertension, triglycerides ≥ 150 mg/dL, HDL-cholesterol < 40 mg/dL (men) or <50 mg/dL (women), and fasting plasma glucose ≥ 100 mg/dL. Framingham risk score (FRS) was calculated based on the National Cholesterol Education Program.

### 2.3. ABI Measurements

BP cuffs were applied to both arms and both ankles, and systolic BP was measured twice at each side using 8 MHz Doppler pen probe and a pocket ultrasonic Doppler flow detector (Microem DV-10). ABI was first calculated according to the AHA definition by dividing the higher systolic pressure of the right and left ankle by the higher systolic pressure of both arms. The lowest ABI value was selected, and the presence of PAD was defined by an ABI value of less than 0.9. The modified ABI was calculated by considering the lower instead of the higher of the two ankle pressures.

### 2.4. Biochemical Evaluation and Electrocardiogram

 After 12-hour period of fasting, venous blood was collected from participants to measure total cholesterol, triglycerides (TG), high-density lipoprotein cholesterol (HDL-c), glucose, creatinine, and uric acid (enzymatic methods). The low-density lipoprotein cholesterol (LDL-c) level was calculated by the Friedewald formula when TG levels were <400 mg/dL. Estimated glomerular filtration rate (GFR) was assessed using Cockroft-Gault formula:

(1) $$\begin{array}{c} \text{GFR}=\left[\frac{\left(140-\text{age}\right)\times \text{weight}\;}{\left(\text{creatinine}\times 72\right)}\right]\left(\times \;0.85\;\text{if}\;\text{female}\right). \end{array}$$

Standard electrocardiogram (ECG) was obtained, and left ventricular hypertrophy was diagnosed according to the Cornell criteria when the sum of R-aVL and S-V3 was at least 28 mm for men or 24 mm for women.

### 2.5. Statistical Analysis

All data are expressed as mean ± SEM, unless otherwise stated. Unpaired Student's *t* test was used to compare means. The chi-square test was used to evaluate the association among categorical variables. Pearson's coefficients were calculated to identify correlation between clinical variables and ABI. All analyses were conducted in Prism software (GraphPad version 5.0). Statistical difference was accepted at a *P* value of less than 0.05.

## 3. Results

**Figure 1:**
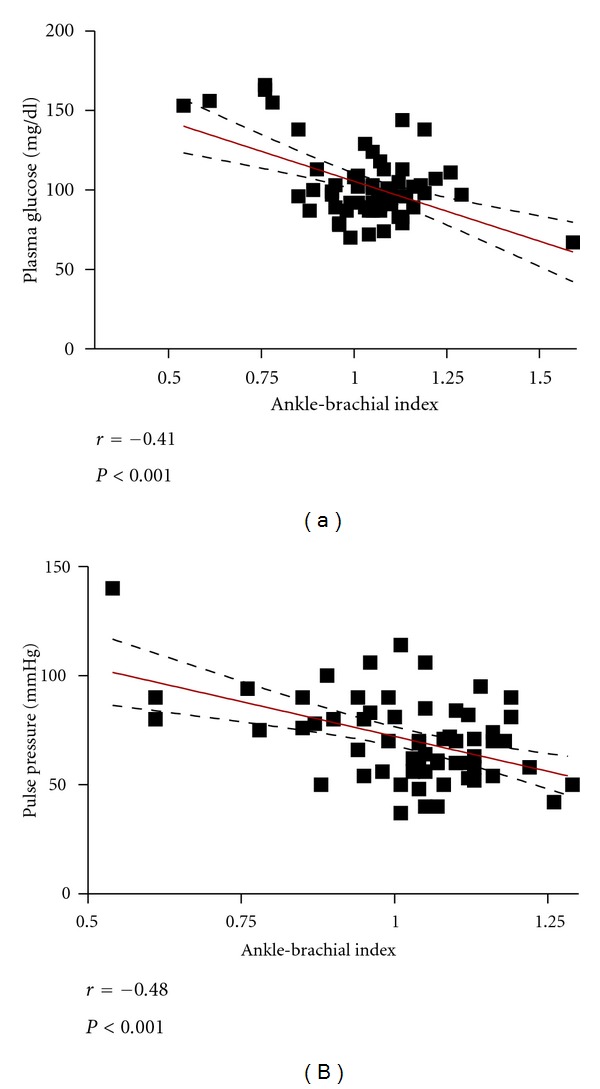
Correlation of ankle-brachial index (ABI) with fasting glucose (a) and with pulse pressure (b).

**Figure 2:**
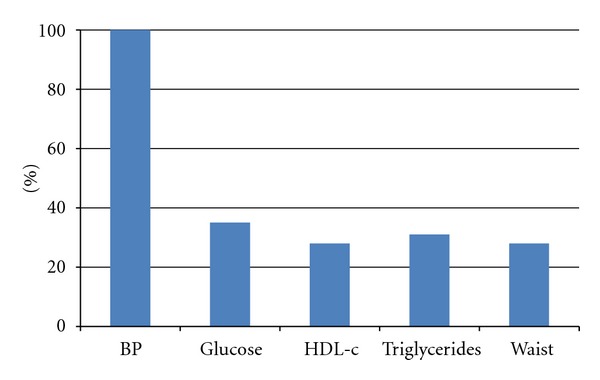
Proportion of criteria for metabolic syndrome in the study population.

The mean age of our sample (*n* = 65) was 73 years, ranging from 65 to 90 years, and mostly composed by women (76%) and white (62%) subjects (Table 1). A low ABI (≤0.9) occurred in 12 (18%) subjects. There was a significantly higher prevalence of diabetes mellitus, cardiovascular disease, metabolic syndrome, and elevated cardiovascular risk by FRS in the group with low ABI (Table 2). Considering all patients in both groups, there was a moderate inverse correlation between fasting glucose and ABI (Figure 1(a)). Among criteria that defined metabolic syndrome, the most important factor in these older hypertensive patients was fasting glucose (Figure 2).

Elderly hypertensive patients with low ABI also presented higher systolic BP and pulse pressure. In addition, when the whole sample was considered for analysis, there was an inverse correlation between pulse pressure and ABI (Figure 1(b)). Patients in the group with low ABI were in use of a higher number of medications, including antihypertensive drugs (Table 3). 

All patients enrolled in this study also had their ABI measured by the modified definition. In this context, the prevalence of low ABI was 32% (21 patients), which represented an additional PAD detection of 14%. The frequency of patients presenting diabetes mellitus, cardiovascular disease, metabolic syndrome, and elevated cardiovascular risk remained higher in the new low ABI group. Likewise, pulse pressure, total number of medications, and antihypertensive drugs in use were significantly higher among these patients. Differently when using ABI by the AHA definition, smoking status and a higher level of LDL-cholesterol became more prevalent in the group with a low ABI by the modified definition (Tables 2 and 3).

## 4. Discussion 

To the best of our knowledge, this is the first study to compare the ankle-brachial index using the higher and the lower of the two ankle pressures on a sample focused in elderly subjects. The present study is also the first to evaluate PAD in elderly persons taking into account their clinical heterogeneity. This was carried out by a comprehensive geriatric assessment that included only subjects without great cognitive impairment or independent for daily living activities. Thus, this study population represents an outpatient group of elderly literate people with a small number of falling events, disturbances of motion and balance, urinary incontinence, depressive symptoms, and with great social support. This should be take into account since the clinical and geriatric profile differs significantly among outpatients and inpatients. 

In these older subjects, the prevalence (18%) of low ABI by current definition was remarkably similar to other studies [9, 13, 14]. Murabito et al. have found a low ABI among 20% of their elderly cohort with a mean age of 80 years [9]. Likewise, ABI < 0.9 was associated with diabetes, cardiovascular disease, and metabolic syndrome. In contrast to other studies, our results demonstrated no association between PAD and smoking status. Although it is well known that smoking significantly increases PAD risk in elderly people [27, 28], we believe that this lack of association could be related to the lower number of current smokers. 

All individuals with a low ABI by the current definition had a higher cardiovascular risk by FRS, which is additional data to confirm the relationship of ABI with cardiovascular events. Although considering the limited accuracy of FRS, since it can overestimate risk in low-risk populations and underestimate risk in high-risk individuals, the questionnaire is still the reference standard for assessing cardiovascular risk worldwide [29]. 

Higher level of systolic BP and widening of pulse pressure were also showed to be more prevalent among those with ABI ≤ 0.9. These were expected results since there is increasing systolic and falling diastolic BP related to ageing [30, 31]. These findings also confirm other studies that reported the association between pulse pressure and PAD [32, 33]. On the other hand, no difference was observed regarding systolic hypertension diagnosis, which may reflect a stronger association with the magnitude of BP level. The lack of association of ABI and left ventricular hypertrophy probably is more associated with the limited accuracy of electrocardiography than with the real pathological myocardial injury. 

The current study also observed the relationship between low ABI and the number of drugs in use, considering both all drugs and only the antihypertensive medications. These data suggest that ABI ≤ 0.9 could be associated with a great number of morbidities and a difficulty to treat hypertension. Furthermore, acetylsalicylic acid and calcium channel antagonists were more widespreadly prescribed to our patients with low ABI, which may reflect a greater prevalence of cardiovascular diseases and more resistant hypertension in this group. 

No difference was found in relation to glomerular filtration rate. This result contrasts with other papers where low ABI was associated with an impairment in kidney function and higher levels of creatinine [34, 35]. Evaluating outpatient subjects, O'Hare et al. have found that ABI lower than 0.9 was more prevalent than ABI of 1.0 or higher in patients with a 50% increase in serum creatinine during a 3-year follow-up. 

A lack of association was also observed between uric acid and ABI. Although most data currently point out a relationship between increased uric acid and low ABI [36, 37], no study exclusively evaluated elderly people. No difference was noted about total cholesterol levels, which is similar to other studies [5, 38, 39]. Indeed, it is not known whether cholesterol is really associated with PAD. On the other hand, reduced HDL-cholesterol levels were more frequent among hypertensive elderly subjects with low ABI, which probably reflects the higher prevalence of metabolic syndrome in these patients. 

Low ABI by the modified definition detected nine additional patients with PAD. This was an expected finding since the method sensitivity was changed. These data were also similar to those reported by Espinola-Klein et al. who indicated 10.8% greater detection of newer subjects with PAD by the modified ABI [40]. Low ABI by the modified definition was also associated with a greater prevalence of diabetes, cardiovascular diseases, and metabolic syndrome; elevated risk by Framingham score; higher levels of systolic BP, pulse pressure, and number of total and antihypertensive drugs in use. These findings suggest that modified definition at least has the same value of current definition. 

Besides detecting more patients with PAD, the most important difference obtained from the modified ABI definition was the association with smoking status and LDL-cholesterol. By detecting a relationship between smokers and PAD, this could be the first result to suggest a greater value to the modified definition. The same interpretation could be applied to LDL-cholesterol, although this association is much more questionable than with smoking. 

Concerning cardiovascular risk, it should be noticed that 3 of the 21 subjects with low ABI had not high risk by FRS. These patients did not have diabetes, a positive smoking status, or any cardiovascular disease. Otherwise, they were obese and had metabolic syndrome and one presented left ventricular hypertrophy by electrocardiogram. Although additional evaluation is necessary in these patients to assess the presence of atherosclerosis, these findings suggest that ABI measurement, especially by a modified definition, could improve the accuracy of cardiovascular risk prediction beyond FRS in the elderly. Recently, a meta-analysis, including persons with age between 47 and 78 years, concluded that ABI measurement could improve the accuracy of cardiovascular risk prediction beyond FRS. Inclusion of the ABI in cardiovascular risk stratification using the FRS resulted in reclassification of the risk category and modification of treatment recommendations in approximately 19% of men and 36% of women [36]. 

Some limitations of the present study should be considered. Firstly, this sample cannot be extrapolated to patients older than 80 years or living in nursing homes and other institutions. Secondly, when electing just subjects independent for daily living activities and without significant cognitive impairment, a survival bias is obtained since more fragile patients were excluded from analysis. Furthermore, we have common limitations with cross-sectional studies when compared to cohort studies, mainly the difficulty in establishing cardiovascular risk. These issues will be reduced as additional data from the original cohort are obtained.

## 5. Conclusion 

In conclusion, the incorporation of clinical markers of asymptomatic atherosclerosis such as ABI can improve prediction of healthy older individuals at high risk of cardiovascular disease. This is the first study to compare two ABI definitions focusing on elderly hypertensive patients taking into account the typical heterogeneity of this group. Considering that a simple modification of the index, using the lower instead of the higher ankle pressure, would identify more patients at risk, the use of a modified ABI calculation should be considered for cardiovascular risk prediction in all hypertensive outpatients older than 65 years. 

## Figures and Tables

**Table 1:** General characteristics of the study population.

Characteristic	*n* (%)
Age (years)	
65–69	19 (30%)
70–74	23 (35%)
75–80	13 (20%)
>80	10 (15%)
Male	13 (24%)
White	40 (62%)
Educational status	
Illiterate	6 (9%)
Literate	59 (91%)
Falling events (>3 in the last year)	1 (1.5%)
Sensitive impairment	
Visual	25 (38%)
Hearing	8 (12%)
Depressive symptoms (GDS > 5)	12 (18%)
Urinary incontinence	4 (6%)
Disturbances of motion and balance	1 (1.5%)
Polypharmacy	19 (29%)
Social support	63 (97%)

GDS: geriatric depression scale.

**Table 2:** Clinical and laboratorial characteristics of the groups with normal (>0.9) and low (≤0.9) ankle-brachial index (ABI) obtained by AHA/TASC II standardization and by modified definition.

Characteristic	AHA definition			Modified definition		
	ABI > 0.9 (*n* = 53)	ABI ≤ 0.9 (*n* = 12)	*P* value	ABI > 0.9 (*n* = 44)	ABI ≤ 0.9 (*n* = 21)	*P* value
Age, years	73.5 ± 0.8	72.1 ± 1.3	NS	73.4 ± 0.9	72.9 ± 1.1	NS
Body mass index, kg/m^2^	27.1 ± 0.6	27.5 ± 1.6	NS	26.7 ± 0.7	28.0 ± 1.0	NS
Waist circumference, cm	92.3 ± 1.9	94.7 ± 3.6	NS	91.3 ± 2.0	95.6 ± 2.6	NS
Metabolic syndrome, *n* (%)	22 (41%)	9 (75%)	<0.05	17 (38%)	14 (67%)	<0.05
CV diseases, *n* (%)^†^	5 (9%)	7 (58%)	<0.001	3 (6%)	9 (42%)	<0.001
Diabetes, *n* (%)	7 (13%)	10 (83%)	<0.01	9 (20%)	6 (29%)	<0.05
Smoker, *n* (%)	11 (21%)	4 (33%)	NS	9 (20%)	6 (29%)	<0.05
Cardiovascular risk						
(i) FRS ≥ 20%, *n* (%)	20 (38%)	12 (100%)	<0.001	16 (36%)	18 (86%)	<0.001
(ii) FRS < 20%, *n* (%)	33 (62%)	0 (0%)	<0.001	28 (63%)	3 (14%)	<0.001
LV hypertrophy, *n* (%)	13 (24%)	5 (41%)	NS	10 (23%)	8 (38%)	NS
GFR, mL/min/1.73 m^2^	68 ± 3	66 ± 10	NS	69 ± 3	68 ± 6	NS
Fasting glucose, mg/dL	94 ± 3.5	139 ± 3.0	<0.05	98 ± 2	120 ± 9	<0.05
Total cholesterol, mg/dL	211 ± 5	227 ± 17	NS	209 ± 6	223 ± 10	NS
LDL-cholesterol, mg/dL	130 ± 5	146 ± 20	NS	124 ± 5	154 ± 13	<0.05
HDL-cholesterol, mg/dL	53 ± 2	42 ± 2	<0.05	54 ± 2	43 ± 2	0.01
Triglycerides, mg/dL	139 ± 10	167 ± 24	NS	141 ± 11	151 ± 16	NS
Uric acid, mg/dL	5.5 ± 0.2	5.2 ± 0.4	NS	5.6 ± 0.2	5.3 ± 0.3	NS
ABI, arbitrary units	1.07 ± 0.01	0.81 ± 0.02	<0.01	1.08 ± 0.01	0.75 ± 0.03	<0.001

Data are expressed as mean ± SEM or *n* (%) when indicated. ABI: ankle-brachial index; CV: cardiovascular; FRS: Framingham risk score; LV: left ventricular; GFR: glomerular filtration rate; LDL: low-density lipoprotein; HDL: high-density lipoprotein; NS: nonsignificant. ^†^Include coronary heart disease: stroke, or transitory ischemic accident.

**Table 3:** Blood pressure levels and drugs in use in normal and low ankle-brachial index (ABI) groups divided by American Heart Association (AHA) definition and by modified definition.

Variable	AHA definition			Modified definition		
	ABI > 0.9 (*n* = 53)	ABI ≤ 0.9 (*n* = 12)	*P* value	ABI > 0.9 (*n* = 44)	ABI ≤ 0.9 (*n* = 21)	*P* value
Systolic BP, mmHg	152 ± 3	169 ± 9	<0.05	150 ± 3	164 ± 6	<0.05
Diastolic BP, mmHg	84 ± 1	83 ± 4	NS	83 ± 1	85 ± 3	NS
Pulse pressure, mmHg	67 ± 2	87 ± 7	<0.01	67	78	<0.05
Total of drugs in use	3.3 ± 0.3	6.3 ± 1.1	<0.001	3.2 ± 0.3	5.1 ± 0.7	<0.01
(i) Acetylsalicylic acid	13 (24.5%)	8 (66.5%)	<0.01	9 (20.5%)	12 (57.1%)	<0.01
(ii) Statins	10 (19%)	3 (25%)	NS	7 (15.9%)	5 (23.8%)	NS
Antihypertensive drugs	1.9 ± 0.1	2.6 ± 0.4	<0.05	1.9 ± 0.1	2.4 ± 0.2	<0.05
(i) ACE inhibitors/ARB	30 (56%)	10 (83%)	NS	26 (59.1%)	14 (66.6%)	NS
(ii) Calcium antagonists	11 (21%)	8 (67%)	<0.01	9 (20.5%)	10 (47.6%)	<0.05
(iii) Beta-blockers	12 (23%)	5 (41%)	NS	9 (20.5%)	8 (38.1%)	NS
(iv) Diuretics	25 (48%)	5 (41%)	NS	21 (47.7%)	9 (42.9%)	NS

Data are expressed as mean ± SEM or *n* (%) when appropriate. BP: blood pressure; ACE: angiotensin converting enzyme; ARB: angiotensin receptor blockers; NS: nonsignificant.
